# Editorial Correspondence

**Published:** 1873-01

**Authors:** W. F. Westmoreland

**Affiliations:** New York


### EDITORIAL AND MISCELLANEOUS.

### EDITORIAL CORRESPONDENCE.

New York, December 21, 1872.

Dear Journal,—Since my arrival in this great medical as well as commercial center, I have met many medical friends, and have seen and heard much to interest me. As is usual with me, my first visit after my arrival was to that *great* and *good* man, Dr. J. Marion Sims. I found the Doctor looking a little worn from the arduous professional labors of the year, but in excellent health and spirits, and although sixty years of age, would readily pass for forty-five.

With him, I visited the Woman's Hospital, which as usual, I found filled with interesting cases. Among others, a case of retroversion of the uterus, with a displacement of the right ovary, for the relief of which Dr. Sims proposed a new and novel operation. The lady is thirty years of age, and has for many years been a great sufferer. She is never free from pain. The displaced ovary is excessively irritable, the least pressure producing an amount of suffering not to be endured. When the womb is placed in its normal position, she is comparatively comfortable, but every instrument or appliance yet adopted has compressed or otherwise irritated the displaced ovary to the extent of producing a train of symptoms requiring its withdrawal.

Up to a few weeks ago he had proposed to perform what my friend Dr. Battey, of Rome, Georgia, has named "normal ovariotomy;" but not as Dr. Battey has recently performed it—removing both ovaries by a section through the abdominal walls—but he proposed to remove the displaced ovary through the posterior cul-de-sac of the vagina. The operation now proposed, and which will be performed in a few days, is the attachment of the fundus of the womb to the abdominal walls. Dr. Sims has had constructed an instrument the length and shape of a uterine sound, which is armed with a stout needle which may be projected at will. This instrument is introduced into the womb and the organ elevated until the fundus comes in contact with the walls of the abdomen, and when at the proper point, the needle is projected from the point of the instrument through the abdominal walls, and when thus projected is threaded with a silver wire, and then withdrawn through the womb and vagina—thus passing a silver wire through the walls of the abdomen, the walls of the womb at the fundus into the cavity, through cervical canal and vagina to os externum—one end of the wire passing out through the abdominal walls, and the other end through the vagina. A button or cup-shaped appendage of some material, non-irritating, is attached to the vaginal end of the wire, and by traction of the end passing out through the abdominal walls, the button or cup-shaped attachment is drawn into the vagina and in contact with the neck and mouth of the womb, and the traction continued until the womb is elevated. The fundus is thus brought in contact with the walls of the abdomen, and held in that position by a second button or fixture to the end of the wire passing through the abdominal walls—the object being to hold the womb in this position until sufficient adhesions have formed to prevent displacement. The operation has never been performed, but as soon as the instruments are perfected, which will be in the next few days, the operation will be made, without, as Dr. Sims thinks, any risk to life. In my next letter, I hope to be able to give the operation in detail, with results.

The Woman's Hospital, which is a monument to the energy and enterprise of Dr. Sims, has, since he left for Europe in 1861, been under the control of Dr. Emmett as Surgeon-in-Chief, until a few months ago, when the Trustees divided the Hospital into four wards or sections, and made three other appointments, thus dividing the honors and labors. The new operators are Drs. Sims, Thomas and Peasley; so that the Woman's Hospital can boast of four of the most prominent gynæcologists of not only this country, but Europe.

The medical colleges in this city are all in a flourishing condition, the classes larger than any session since the war.

The Bellevue Hospital Medical College has about five hundred, the College of Physicians and Surgeons three hundred and sixty to seventy, and the University Medical College about two hundred. It will be seen that the University, which is the oldest school, and at one time the most popular, the size of its class doubling that of any other school in the city, has now the smallest class.

Some of the causes which conspired to produce this, we propose to discuss in our next.

Truly, W. F. WESTMORELAND.

